# The median age of a city’s residents and population density influence COVID 19 mortality growth rates: policy implications

**DOI:** 10.1186/s13584-022-00541-w

**Published:** 2022-09-12

**Authors:** Yuval Arbel, Yifat Arbel, Amichai Kerner, Miryam Kerner

**Affiliations:** 1 Sir Harry Solomon School of Economics and Management, Western Galilee College, 2412101 Acre, Israel; 2 Department of Mathematics, Bar Ilan University, Ramat Gan, Israel; 3 School of Real Estate, Netanya Academic College, 4223587 Netanya, Israel; 4 The Ruth and Bruce Rappaport Faculty of Medicine, Technion, Israel Institute of Technology, 3525422 Haifa, Israel; 5 Department of Dermatology, HaEmek Medical Center, 1834111 Afula, Israel

**Keywords:** COVID-19, Mortality

## Abstract

**Background:**

SARS-CoV-2 is an infectious virus, which has generated a global pandemic. Israel was one of the first countries to vaccinate its population, inaugurating the program on December 20, 2020. The objective of the current study is to investigate the projected daily COVID19 mortality growth rate with higher median age and population size of cities under two scenarios: with and without the BNT162b2 Pfizer vaccination against the SAR-COV2 virus.

**Methods:**

This study employs a panel data-set. We follow the COVID19 mortality growth rate in each of the 173 Israeli cities and towns starting from March 21, 2020 (10 days after the first documentation of COVID19 cases in Israel) until September 21, 2021, where the BNT162b2 Pfizer vaccinations were available starting from December 20, 2020.

**Results:**

Referring to the median age of municipal residents, findings suggest that the BNT162b2 Pfizer vaccinations attenuate the rise in anticipated daily mortality growth rate for cities and towns in which the median population age is 30 years old (the range in median age among the residents in the municipalities surveyed is 11–41 years). Moreover, referring to population size of cities, findings demonstrate that while under the scenario without vaccination, the daily mortality growth rate is anticipated to rise, under a comparable scenario with vaccination, daily mortality growth rate is anticipated to drop.

**Conclusions:**

In crowded cities, where the median age is high, two perspectives of early and intensive public policy interventions are clearly required. The first perspective is extensive medical treatment, namely, extension of availability of medical physical and online services; dispensing designated medications; expansion of hospitalization facilities and information services particularly to susceptible populations. All measures will be taken with attention to age accessibility of these means. The second perspective is prevention via establishment of testing and vaccination complexes; elevation of dedicated health services, generating selective lockdowns; education for increasing awareness to social distancing, wearing masks and other preventive means.

## Background

The objective of the current study is to investigate the projected daily COVID19 mortality growth rate with higher median age and population size of cities under two scenarios: with and without the BNT162b2 Pfizer vaccination against the SAR-CoV2 virus. This study employs a panel dataset. We follow the COVID19 mortality growth rate in each of the 173 Israeli cities and towns starting from March 21, 2020 (10 days after the first documentation of COVID19 cases in Israel) until September 21, 2021, where the BNT162b2 Pfizer vaccinations were available starting from December 20, 2020.

Since the beginning of the global vaccination campaign against SARS-CoV2 virus, substantial data concerning the effectiveness, safety and adverse effects of the vaccinations have been collected.^1^ As of December 2, 2021, 54.6% of the world’s population has received at least one dose of a COVID-19 vaccine. 8.07 billion doses have been administered globally, and 23.89 million are currently administered each day. Yet, only 3% of people in low-income countries have received at least one dose. (see: https://ourworldindata.org/covid-vaccinations) [2].

Indeed, COVID19 is one of the remarkable pandemics in the last century (World Health Organization [3–5]. The mortality rate is one of the major complications of the pandemic. As of June 5, 2022, the official accumulated COVID19 deaths worldwide totaled 6.3 million persons, compared to 35 million deaths from the Spanish flu of 1918–1921, where adjustment to current world population yields 150 million deaths [3]. Yet, given the access death toll (i.e., the gap between the number of deaths in a specific region during a given time period, regardless of cause, and how many deaths would have been expected if a disease outbreak had not occurred), the estimated COVID19 death toll might rise fourfold to 16 million deaths with 95% confidence interval of between 9.9 million and 18.6 million (The Economist, October 8, 2021) [6].^2^ In the United States, the official accumulated number of COVID19 deaths (1 Million – as of June 5, 2022) exceed the number of deaths during the 1918–1919 Spanish flu pandemic (AP News, September 21, 2021) [7]. Yet, in Israel, considering the access deaths, the estimated death toll might be smaller than the official death toll (The Economist, October 8, 2021) [6].

Referring to the COVID19 pandemic, Israel provides an interesting case study. Three salient features of Israel are: (1) an accelerated urbanization process and non-uniform distribution of population densities, which, in turn, might increase the spread of the pandemic [8, 9], (2) disparities in household income and socio-economic ranking of municipalities (cities and towns) [27]; and (3) the early initiation of a nationwide vaccination campaign leading to the full vaccination (i.e., receipt of two vaccine doses) of more than half the population by the end of March 2021 [10, 11]. There are currently three vaccine types in Israel: (1) Pfizer (approved on December, 2020), (2) Moderna (approved on August, 2021) and (3) Astra-Zenika (approved on September, 2021). Yet, the majority of the Israeli population received the Pfizer vaccine.

Israel has a relatively high per capita income (42,403 US Dollars per-capita); high income inequality (Gini coefficient of 35.5% in 2018) and is also characterized by an advanced health system and with universal health-care insurance available by law.^3^ To address inequalities in availability and access to health care, legislation providing for universal health-care insurance for all Israeli citizens was passed in 1995 [12]. This law provides a broad basket of high-quality preventive, curative, and rehabilitation health-care services. Overall, the health status has improved steadily in recent decades. Between 1975 and 2014, life expectancy in Israel steadily increased and is currently above the average life expectancy for the Organization for Economic Co-operation and Development (OECD) countries [13].

In another context (i.e., obesity pandemics), Muhsen et al. [13] notes health disparities related to income and ethnic origin (lower life expectancy among Israeli Arab compared to Jewish Israelis), and the extent of supplementary health insurance programs. The aim of these programs is to reduce inequalities in health care by a combination of policies, including reduced copayments, a focus on at-risk populations, and intervention measures adapted to language, culture, literacy, and comprehension levels. For example, Ethiopian cultural mediators were employed in clinics in neighborhoods with large numbers of Ethiopian immigrant residents. In Arab villages, personal nurses were introduced into local clinics as case managers for diabetes patients.

Dagan et al. [14] tested the effectiveness of the vaccine in Israel after the first and second doses. The authors generated a matched-paired sample of 596,618 vaccinated and 596,618 unvaccinated individuals with similar characteristics. They found at days 14–20 after the first dose a 46% effectiveness in terms of documented infections, a 57% effectiveness in terms of symptomatic COVID19 cases; a 74% effectiveness in terms of hospitalizations and a 62% effectiveness in terms of severe cases.^4^ Seven days after the second dose, the vaccine effectiveness rises to a 92% effectiveness in terms of documented infections; a 94% effectiveness in terms of symptomatic COVID19 cases; a 87% effectiveness in terms of hospitalizations and a 92% effectiveness in terms of severe cases.

Referring to the median age of municipal residents, findings suggest that the BNT162b2 Pfizer vaccinations attenuate the pace of anticipated daily mortality growth rate *rise* for 30 years of median age (the range in median age among the residents in the cities surveyed is 11–41 years). The literature indeed demonstrates that age is a risk factor for COVID19 infection and mortality (e.g., [5, 15, 16].

Moreover, referring to population size and population density of municipalities, findings demonstrate that while under the scenario without vaccination, the daily mortality growth rate is anticipated to *rise*, under the scenario with vaccination, daily mortality growth rate is anticipated to *drop*. Given the elevated number of interactions in more crowded cities (e.g., [9, 17, 18], the vaccination against SARS-COV2 virus might prove to be particularly important in larger, denser cities.

The remainder of this article is organized as follows. “Descriptive Statistics” Section provides the descriptive statistics. “The Structural Model” Section gives the methodology and “Results” Section, the results. Finally, “Discussion” Section provides discussion and “Conclusions” Section concludes and summarizes.

## Methods

### Descriptive statistics

**Fig. 1:**
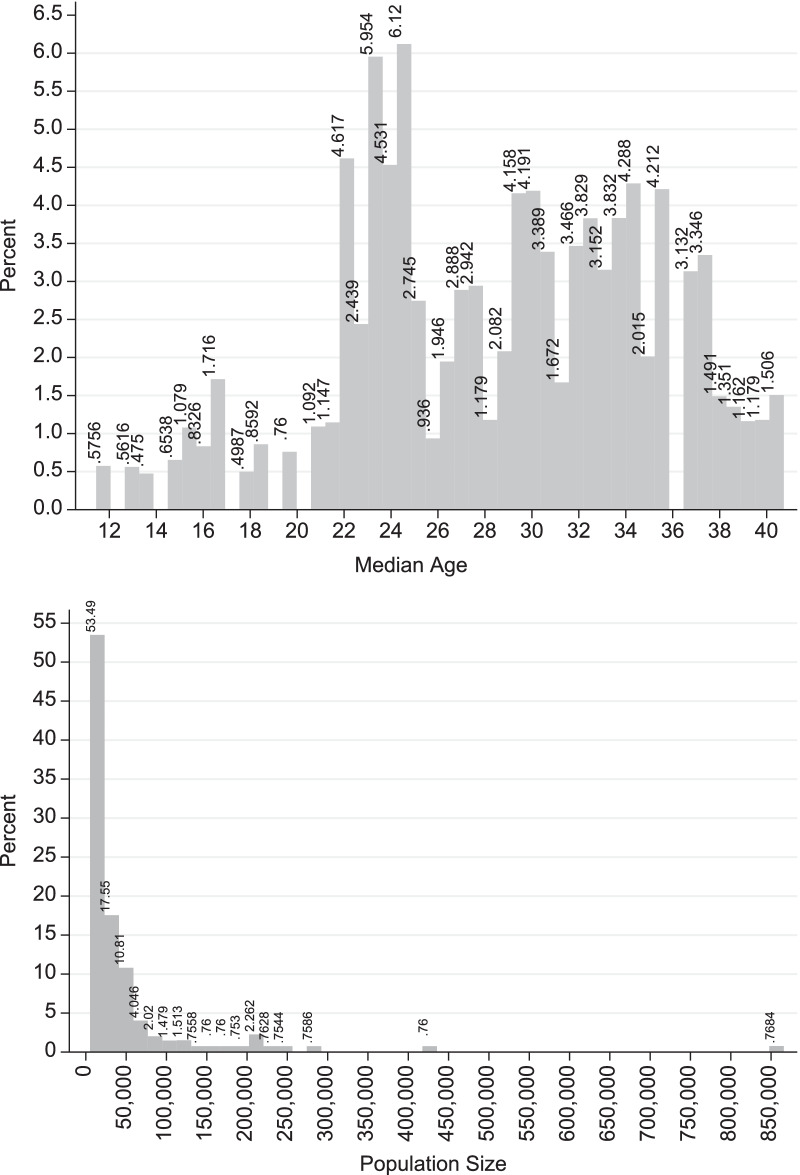
Histograms: median age of population and population size

This section presents the variables that are employed later in the empirical model. Figure 1 provides the histograms of the median age and the population size of the cities and towns. Table 1 gives the descriptive statistics of the variables incorporated in the empirical model. The dataset employs in this study refers to 173 Israeli municipalities ($i=\mathrm{1,2},3,\cdots ,173$) and 550 days ($t=\mathrm{11,12,13},\cdots ,560$), where $i\times t=\mathrm{71,580}$. *t* is defined as the daily time variable from 1 (= March 11, 2020; the first documentation of COVID19 cases) to 560 (= September 21, 2021). For convenience, throughout the article the indices *i* and *t* are interchangeably used or deleted.

**Table 1:** Descriptive Statistics

Variable	Description	Obs	Mean	Std. dev.	Min	Max
*t*	Daily time variable from 1 (= March 11, 2020; the first documentation of COVID19 cases) to 560 (= September 21, 2021)	71,580	342.57	134.79	11	560
$\Delta ln(Cum\_Deaths)=ln{(Cum\_Deaths)}_{t}-ln{\left(Cum\_Deaths\right)}_{t-1}$	Approximated daily mortality growth rate from SARS-COV2 virus in each of the 173 Israeli municipalities where $Cum\_Deaths$ is the accumulated number of deaths	71,580	0.0013	0.0074	0	0.2113
*MedianAge*	Median age of municipal residents in years	71,580	28.71	6.37	11.44	40.73
*PopulationSize*	Population size of the municipality	71,580	51,214.85	95,698.34	5,232	865,721
*Dum_vaccine*	1 = post-vaccine era (December 20, 2020-September 21, 2021); 0 = pre-vaccine era (March 21, 2020-December 19, 2020)	71,580	0.6542	Irrelevant	0	1
*Population*_*density*	Population per Square Kilometer	63,555	3,558	3,699	169	26,512
*Lockdowns*	1 = Lockdowns; 0 = otherwise	71,580	0.1733	Irrelevant	0	1
*Holidays*	1 = Holidays; 0 = otherwise	71,580	0.1154	Irrelevant	0	1

The dataset refers to 173 Israeli municipalities ($i=\mathrm{1,2},3,\dots ,173$) and 550 days ($t=\mathrm{11,12,13},\dots ,560$), where $i\times t=\mathrm{71,580}$

The dependent variable $\Delta ln(Cum\_Deaths)$ equals $ln{(Cum\_Deaths)}_{t}-ln{\left(Cum\_Deaths\right)}_{t-1}$ and reflects the approximated daily mortality growth rate from SARS-COV2 virus in each of the 173 Israeli cities and towns (*Cum_Deaths* is the accumulated number of deaths). As demonstrated by Johnston and Dinardo (1997: 42–45) [19], and referring to the semi-logarithmic model: $ln{Y}_{t}=\alpha +\beta t$, the coefficient of the variable *t* reflects an approximation to the constant growth rate.^5^ Consequently, $ln{(Cum\_Deaths)}_{t}-ln{\left(Cum\_Deaths\right)}_{t-1}$ reflects the approximated daily change in mortality rate.

The average mortality daily growth rate is 0.13% and the standard deviation is 0.7%. The maximum daily mortality growth rate is 21.13%. This was obtained in Jerusalem on April 10, 2020 (from 17 to 21 dead persons).

The independent variables include: *MedianAge*; *PopulationSize Population_Density*, *Lockdowns*; *Holidays* and *Dum_vaccine*. *Dum_vaccine* equals 1 for the post-vaccine era (December 20, 2020–September 21, 2021); 0 = pre-vaccine era (March 21, 2020–December 19, 2020). According to Table 1, during the sample period, the COVID19 vaccine was available 65% of the time. Referring to *MedianAge* (the median age of city residents in years), the mean median age is 28.71 and the standard deviation is 6.37 years. As Fig. 1 demonstrates, the modal median age is around 24 years (6.12 percent), and the distribution of median age of the residents appears to be uniform.

Referring to *PopulationSize* (population size of the municipality), the mean is 51,215 residents and the standard deviation is 95,698 inhabitants.^6^ As the bottom part of Fig. 1 demonstrates, the population size is skewed to the right (skewness = 5.5685). While 50 percent of the municipalities include 21,611 persons or fewer, and 80 percent of the municipalities include 55,464 persons, the population of only one city is above 865,000 persons (Jerusalem). The minimum is 5,232 persons (Geva Binyamin) and the maximum is 865,721 persons (Jerusalem). Indeed, Israel is characterized by high urbanization levels and non-uniform distribution of population densities, which, in turn, might increase the spread of the pandemic [8, 9].

Referring to the *Population_Density* variable, the sample mean is 3558 and the standard deviation is 3699 persons per square kilometer. The minimum is 169 and the maximum is 26,512 persons per square kilometer.

### The structural model

Consider the following models, each of which is estimated separately:

1 $$\Delta ln{\left(Cum\_Deaths\right)}_{i,t}={\alpha }_{0}+{\alpha }_{1}{PopulationSize}_{i}+{\alpha }_{2}\left({MedianAge}_{i}-11\right)\times t+{\alpha }_{3}{Dum\_Vaccine}_{t}\times \left({MedianAge}_{i}-11\right)\times t+{\alpha }_{4}{Population\_Density}_{i}+{\alpha }_{5}{Lockdowns}_{t}+{\alpha }_{6}{Holidays}_{t}+A{\overrightarrow{\delta }}_{1}+{\in }_{1,i,,t}$$

2 $$\Delta ln{\left(Cum\_Deaths\right)}_{i,t}={\beta }_{0}+{\beta }_{1}{MedianAge}_{i}+{\beta }_{2}{PopulationSize}_{i}\times t+{\beta }_{3}{Dum\_Vaccine}_{t}\times {PopulationSize}_{i}\times t+{\beta }_{4}{Population\_Density}_{i}+{\beta }_{5}{Lockdowns}_{t}+{\beta }_{6}{Holidays}_{t}+A{\overrightarrow{\delta }}_{2}+{\in }_{2,i,,t}$$

and

3 $$\Delta ln{\left(Cum\_Deaths\right)}_{i,t}={\gamma }_{0}+{\gamma }_{1}{MedianAge}_{i}+{\gamma }_{2}{Population\_Density}_{i}\times t+{\gamma }_{3}{Dum\_Vaccine}_{t}\times {Population\_Density}_{i}\times t+{\gamma }_{4}{Population\_Density}_{i}+{\gamma }_{5}{Lockdowns}_{t}+{\gamma }_{6}{Holidays}_{t}+A{\overrightarrow{\delta }}_{3}+{\in }_{3,i,,t}$$

where *i* is the index of cities ($i=\mathrm{1,2},3,\cdots ,173$), *t* is Daily time variable from 1 (= March 11, 2020; the first documentation of COVID19 cases) to 560 (= September 21, 2021);$\Delta ln{\left(Cum\_Deaths\right)}_{i,t}$, the dependent variable, is the approximated daily mortality growth rate from SARS-COV2 virus and $Cum\_Deaths$ is the accumulated number of deaths. The independent variables are:${PopulationSize}_{i}$, ${Population\_Density}_{i},$ ${Lockdowns}_{t}$ and ${Holidays}_{t};$ $\left({MedianAge}_{i}-11\right)\times t$ (base category – pre-vaccine era for the city with a minimum median age of population of 11 years) and ${Dum\_Vaccine}_{t}\times \left({MedianAge}_{i}-11\right)\times t$ (the difference between post and pre-vaccine era) in Eq. (1)^7^;${MedianAge}_{i}$, ${Population\_Density}_{i},$ ${Lockdowns}_{t}$ and ${Holidays}_{t}; {PopulationSize}_{i}\times t$ (base category – pre-vaccine era for the city with a minimum population size of 1 person) and ${Dum\_Vaccine}_{t}\times {PopulationSize}_{i}\times t$ (the difference between post and pre-vaccine era) in Eq. (2); and${MedianAge}_{i}$, ${PopulationSize}_{i},$ ${Lockdowns}_{t}$ and ${Holidays}_{t}; {Population\_Density}_{i}\times t$ (base category – pre-vaccine era for the city with a minimum population density of 1 person per square kilometer) and ${Dum\_Vaccine}_{t}\times {Population\_Density}_{i}\times t$ (the difference between post and pre-vaccine era) in Eq. (3), where ${PopulationSize}_{i}$; ${MedianAge}_{i}$ and ${Population\_Density}_{i}$ are generic and constant across time within the same city; and ${Dum\_Vaccine}_{t}$ ${Lockdowns}_{t}$ and ${Holidays}_{t}$ are constant across cities. Finally, ${\alpha }_{0},{\alpha }_{1},{\alpha }_{2},{\alpha }_{3},$ ${\alpha }_{4},{\alpha }_{5}$; ${\beta }_{0},{\beta }_{1},{\beta }_{2},{\beta }_{3},{\beta }_{4},$ ${\beta }_{5}$ and ${\gamma }_{0},{\gamma }_{1},{\gamma }_{2},{\gamma }_{3},$ ${\gamma }_{4},$ ${\gamma }_{5}$ are parameters, ${\in }_{1,i,,t}$; ${\in }_{2,i,,t}$ and ${\in }_{3,i,,t}$ are the stochastic random disturbance terms, which satisfy all the classical assumptions of the regression model, $A$ is a ($i\times i$) diagonal matrix of dummy variables capturing generic heterogeneity across municipalities and ${\overrightarrow{\delta }}_{1}$; ${\overrightarrow{\delta }}_{2}$ and ${\overrightarrow{\delta }}_{3}$ are the corresponding ($i\times 1$) column vectors.

## Results

**Fig. 2:**
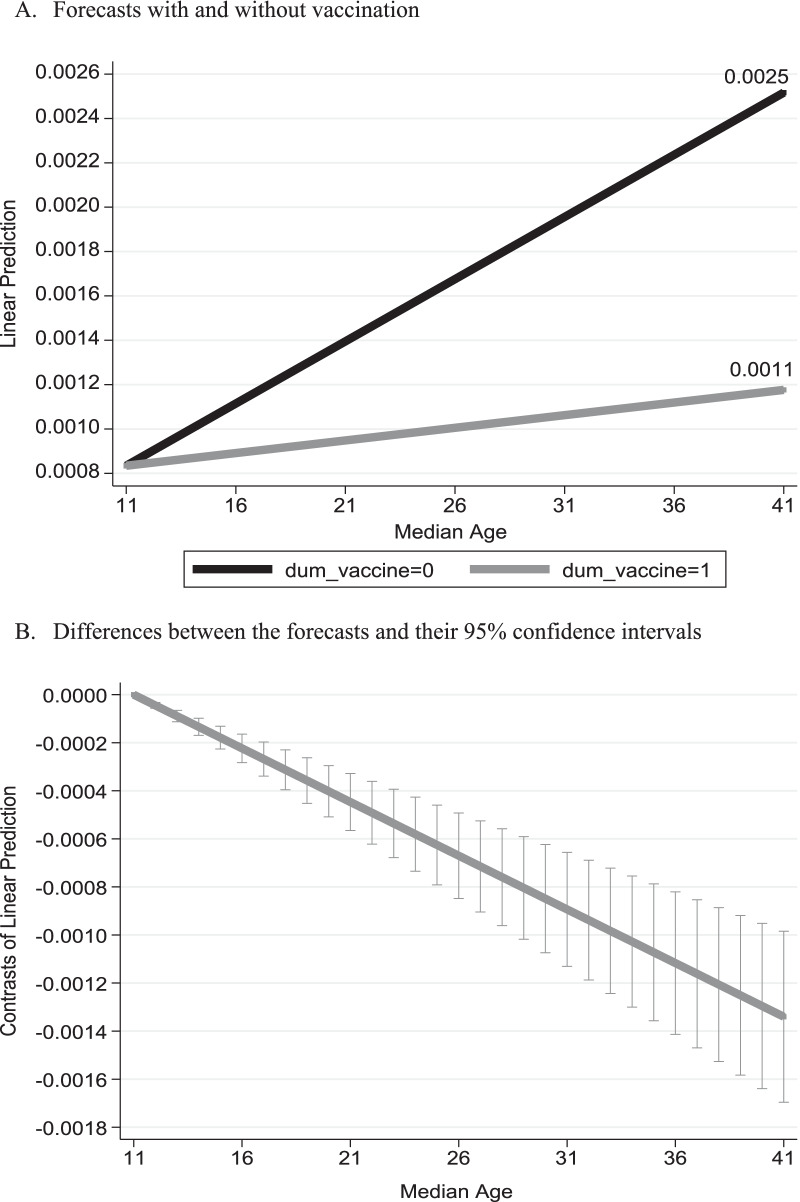
Anticipated mortality daily growth rate versus median age. *Notes*: Based on the outcomes reported in column (1) of Table 2

Based on the regression outcomes obtained from Eq. (1) and reported in Table 2, Fig. 2 refers to the median age of population in the municipalities and describes the anticipated daily mortality growth rate from SARS-COV2 virus under two scenarios: with and without vaccination. The bottom part of Fig. 2 presents the difference between these two scenarios and their 95% confidence intervals.

**Table 2:** Regression analysis

Variables	(1)	(2)	(3)
	$\Delta \mathrm{ln}(Cum\_Deaths)$	$\Delta \mathrm{ln}(Cum\_Deaths)$	$\Delta \mathrm{ln}(Cum\_Deaths)$
Constant	0.000114	− 0.000196	− 0.00142***
	(0.324)	(0.158)	(< 0.01)
PopulationSize	1.41 × 10^–8^***	1.29 × 10^–8^***	1.24 × 10^–8^***
	(< 0.01)	(< 0.01)	(< 0.01)
(MedianAge 11) × .*t*	1.64 × 10^–7^***	2.06 × 10^–7^***	4.28 × 10^–7^***
	(6.68 × 10^–10^)	(< 0.01)	(< 0.01)
Dum_vaccine × (MedianAge 11) × .*t*	3.33 × 10^–8^***	3.88 × 10^–8^***	1.67 × 10^–7^***
	(0.00659)	(0.00315)	(< 0.01)
Population_Density	–	8.80 × 10^–8^***	9.23 × 10^–8^***
	–	(0.000104)	(5.24 × 10^–5^)
Lockdowns	–	–	0.00229***
	–	–	(< 0.01)
Holidays	–	–	− 0.000118
	–	–	(0.191)
Observations	71,580	63,555	63,555
R-squared between estimators	0.6118	0.6372	0.6461
Calculated wald chi^2^ for the regression significance	363.61***	365.25***	1,197.41***
Number of CityCode	173	152	152

Estimation outcomes are based on the empirical model given by Eq. (1). MedianAge = 11 is the minimum age. The R-squared between estimators gives the goodness of fit for the general equation ${\overline{y} }_{i}=\alpha +{\overline{x} }_{i}\beta +{\nu }_{i}+{\overline{\varepsilon }}_{i}$ where ${\overline{y} }_{i}={\sum }_{t}{y}_{it}/{T}_{i}$; ${\overline{x} }_{i}={\sum }_{t}{x}_{it}/{T}_{i}$; ${\overline{\epsilon }}_{i}={\sum }_{t}{\epsilon }_{it}/{T}_{i}$ (the sample mean of cities across time) and ${\nu }_{i}$ reflect generic differences across cities. *p*-values are given in parentheses

***p < 0.01

As can be seen from the figure, the empirical model imposes the same anticipated mortality growth rate under these two scenarios for the municipality with the minimum median age of population of 11 years (0.08%). Under the scenario without vaccination, the daily mortality growth rate is anticipated to *rise* to 0.25% for the municipality whose median age of population of 41 years. Vaccination attenuates this *rise* to only 0.1% for the city with the median age of 41 years. The bottom figure demonstrates the rejection of the null hypothesis of no differences between these two scenarios, and thus stresses the importance of the BNT162b2 vaccine in reducing mortality even for more susceptible groups, i.e., older residents.

**Fig. 3:**
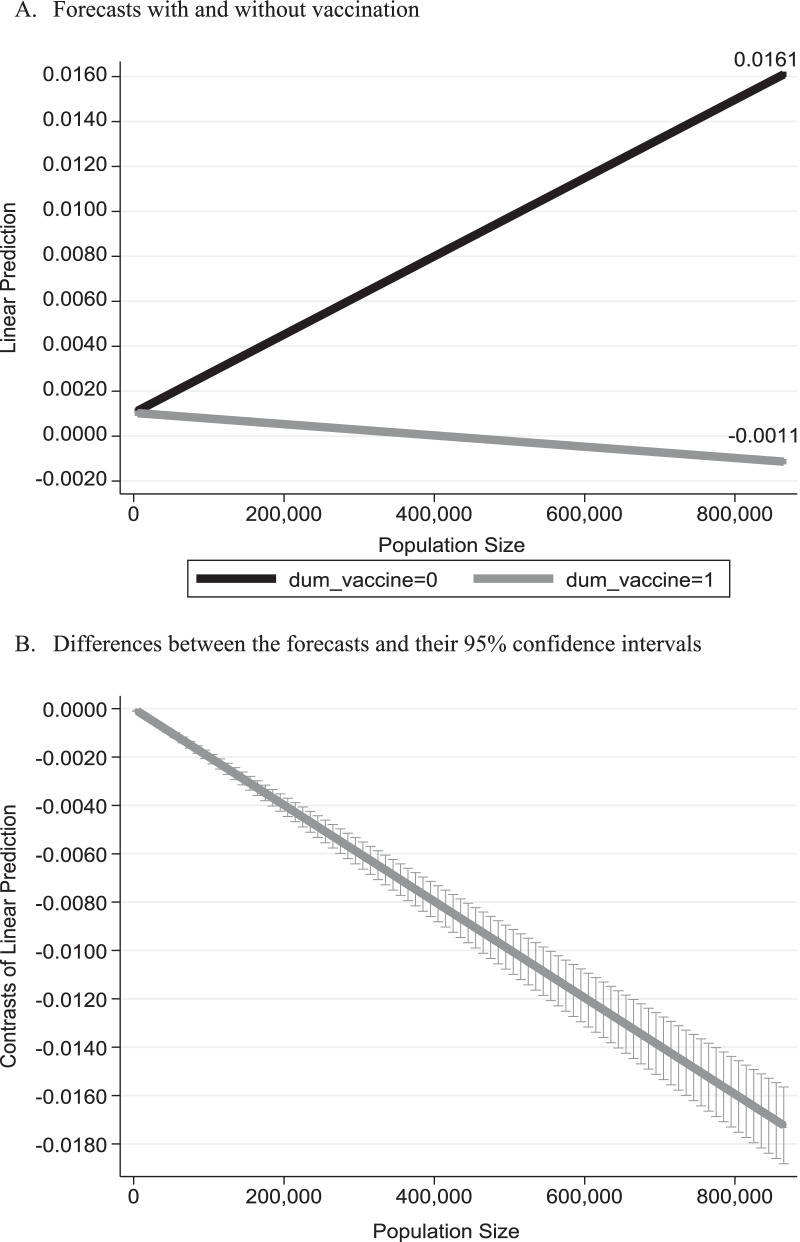
Anticipated mortality daily growth rate versus population size. *Notes*: Based on the outcomes reported in column (1) of Table 3

Figure 3 relates to the population size of municipalities and presents the anticipated daily mortality growth rate from SARS-COV2 virus under two scenarios: with and without vaccination. It is based on the regression outcomes obtained from Eq. (1) and reported in Table 3. The bottom part of Fig. 3 provides the difference between these two scenarios and their 95% confidence intervals.

**Table 3:** Regression analysis

Variables	(1)	(2)	(3)
	$\Delta \mathrm{ln}(Cum\_Deaths)$	$\Delta \mathrm{ln}(Cum\_Deaths)$	$\Delta \mathrm{ln}(Cum\_Deaths)$
Constant	− 0.00150***	− 0.00193***	− 0.00214***
	(8.43 × 10^–6^)	(7.53 × 10^–8^)	(1.55 × 10^–9^)
MedianAge	8.81 × 10^–5^***	8.50 × 10^–5^***	7.95 × 10^–5^***
	(< 0.01)	(< 0.01)	(7.67 × 10^–11^)
PopulationSize × *t*	5.08 × 10^–11^***	4.22 × 10^–11^***	5.19 × 10^–11^***
	(< 0.01)	(< 0.01)	(< 0.01)
Dum_vaccine × .PopulationSize × *t*	− 7.32 × 10^–12^***	− 1.02 × 10^–11^***	− 4.37 × 10^–12^***
	(7.50 × 10^–7^)	(2.06 × 10^–10^)	(0.00679)
Population_Density	–	1.82 × 10^–7^***	1.63 × 10^–7^***
	–	(< 0.01)	(< 0.01)
Lockdowns	–	–	0.00197***
	–	–	(< 0.01)
Holidays	–	–	− 4.14 × 10^–5^
	–	–	(0.642)
Observations	71,580	63,555	63,555
R-squared between estimators	0.318	0.3471	0.4736
Calculated F-value for the regression significance	900.92***	633.38***	557.88***
Number of CityCode	173	152	152

Estimation outcomes are based on the empirical model given by Eq. (2). The R-Squared between estimators gives the goodness of fit for the general equation ${\overline{y} }_{i}=\alpha +{\overline{x} }_{i}\beta +{\nu }_{i}+{\overline{\varepsilon }}_{i}$ where ${\overline{y} }_{i}={\sum }_{t}{y}_{it}/{T}_{i}$; ${\overline{x} }_{i}={\sum }_{t}{x}_{it}/{T}_{i}$; ${\overline{\epsilon }}_{i}={\sum }_{t}{\epsilon }_{it}/{T}_{i}$ (the sample mean of cities across time) and ${\nu }_{i}$ reflect generic differences across cities. *p*-values are given in parentheses

***p < 0.01

As can be seen from the figure, the empirical model imposes the same anticipated mortality growth rate under these two scenarios for a municipality with 1 person (0.10%). While under the scenario without vaccination, the daily mortality growth rate is anticipated to *rise* to 1.61%, under the scenario without vaccination, daily mortality growth rate is anticipated to *drop* to 0.11% for the municipality with 957,600 persons (Jerusalem).^8^ The bottom figure demonstrates the rejection of the null hypothesis of no difference between these two scenarios, and thus reiterates the importance of the BNT162b2 vaccine in reducing mortality, particularly in large cities such as Jerusalem.

**Fig. 4:**
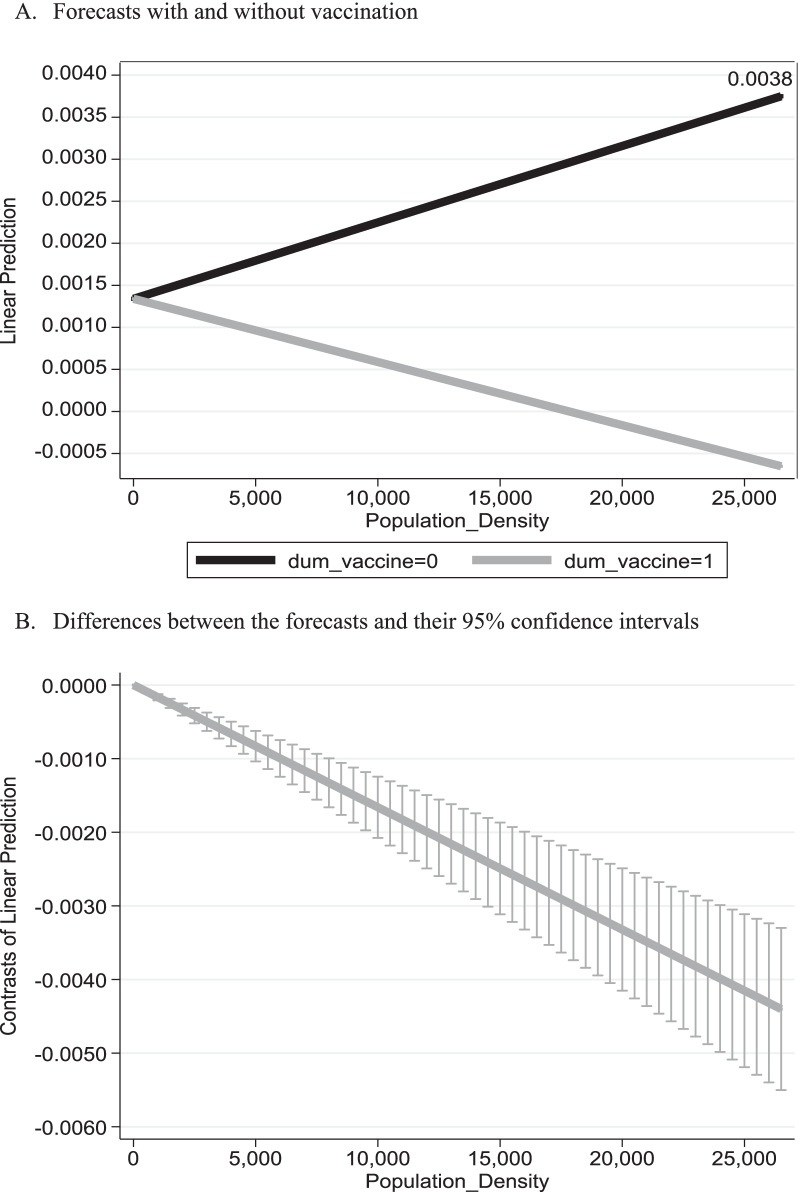
Anticipated mortality daily growth rate versus population density. *Notes*: Based on the outcomes reported in column (1) of Table 4

Figure 4 refers to the population density of municipalities (population per square kilometer) and presents the anticipated daily mortality growth rate from SARS-COV2 virus under two scenarios: with and without vaccination. It is based on the regression outcomes obtained from Eq. (1) and reported in Table 4. The bottom part of Fig. 4 provides the difference between these two scenarios and their 95% confidence intervals.

**Table 4:** Regression analysis

Variables	(1)	(2)	(3)
	$\Delta \mathrm{ln}(Cum\_Deaths)$	$\Delta \mathrm{ln}(Cum\_Deaths)$	$\Delta \mathrm{ln}(Cum\_Deaths)$
Constant	− 0.00105***	− 0.00160***	− 0.00160***
	(0.00208)	(2.74 × 10^–6^)	(3.05 × 10^–6^)
MedianAge	5.93 × 10^–5^***	6.00 × 10^–5^***	6.00 × 10^–5^***
	(6.87 × 10^–7^)	(4.80 × 10^–7^)	(5.21 × 10^–7^)
PopulationSize	1.39 × 10^–8^***	1.29 × 10^–8^***	1.29 × 10^–8^***
	(< 0.01)	(< 0.01)	(< 0.01)
Population_Density × *t*	2.66 × 10^–10^***	5.65 × 10^–10^***	5.67 × 10^–10^***
	(0.00160)	(< 0.01)	(< 0.01)
Dum_vaccine × Population_Density × *t*	− 2.20 × 10^–10^***	− 3.13 × 10^–11^	− 3.12 × 10^–11^
	(3.40 × 10^–9^)	(0.408)	(0.410)
Lockdowns	–	0.00197***	0.00197***
	–	(< 0.01)	(< 0.01)
Holidays	–	–	− 2.62 × 10^–5^
	–	–	(0.769)
Observations	63,555	63,555	63,555
R-squared between estimators	0.6056	0.6583	0.6584
Calculated F-value for the regression significance	639.09***	660.45***	550.43***
Number of CityCode	152	152	152

Estimation outcomes are based on the empirical model given by Eq. (2). The R-Squared between estimators gives the goodness of fit for the general equation ${\overline{y} }_{i}=\alpha +{\overline{x} }_{i}\beta +{\nu }_{i}+{\overline{\varepsilon }}_{i}$ where ${\overline{y} }_{i}={\sum }_{t}{y}_{it}/{T}_{i}$; ${\overline{x} }_{i}={\sum }_{t}{x}_{it}/{T}_{i}$; ${\overline{\epsilon }}_{i}={\sum }_{t}{\epsilon }_{it}/{T}_{i}$ (the sample mean of cities across time) and ${\nu }_{i}$ reflect generic differences across cities. *p*-values are given in parentheses

***p < 0.01

As can be seen from the figure, the empirical model imposes the same anticipated mortality growth rate under these two scenarios for a municipality with 1 person per square kilometer (0.1%). While under the scenario without vaccination, the daily mortality growth rate is anticipated to *rise* to 3.8%, under the scenario without vaccination, daily mortality growth rate is anticipated to *drop* to 0.11% for the Benei-Berak municipality with 26,510 persons per square kilometer. The bottom figure demonstrates the rejection of the null hypothesis of no difference between these two scenarios, and thus reiterates the importance of the BNT162b2 vaccine in reducing mortality, particularly in large cities such as Jerusalem.

At the bottom of the Tables, we report the R-squared of the between estimators and the Wald Chi^2^/F-statistics for the regression significance. All the regressions were found to be highly significant. The R-Squared between estimators gives the goodness of fit for the general equation ${\overline{y} }_{i}=\alpha +{\overline{x} }_{i}\beta +{\nu }_{i}+{\overline{\varepsilon }}_{i}$ where ${\overline{y} }_{i}={\sum }_{t}{y}_{it}/{T}_{i}$; ${\overline{x} }_{i}={\sum }_{t}{x}_{it}/{T}_{i}$; ${\overline{\epsilon }}_{i}={\sum }_{t}{\epsilon }_{it}/{T}_{i}$ (the sample mean of cities across time) and ${\nu }_{i}$ reflect generic differences across cities. In Tables 2, 3 and 4] 61.18–63.72% (31.8–34%) [60.56–65.84%] of the variance of the dependent variable is explained by the independent variables:^9^

## Discussion

Referring to the Israeli context, following the signed vaccine purchase contracts with several pharmaceutical companies, including the Pfizer-BioNTech COVID-19 vaccine, and the FDA emergency use authorization, on December 16, 2020 Israel determined the susceptible population (e.g., people aged 60 years and over, nursing home residents) as the initial target populations to be vaccinated. Rosen et al. [11] As our study indicates, given the steeper rise of anticipated mortality growth rate with the median age of the city in the absence of COVID-19 vaccination, this policy is justified.

According to Rosen et al. [11], Israel has several inherent characteristics, which contributed to the success of the Israeli vaccination campain and thus avoided or reduced the potential negative repercussions associated with COVID-19 mortality growth rate in the absence of vaccination.

Among other features, these include: (1) the small size of the country and the high population density. The Israeli population consists of 9.291 million persons (ICBS media release 438/2020: Population of Israel on the Eve of 2021 (Jerusalem, December 31, 2020), where Israel is also considered to be a highly urbanized nation. According to the Israel Central Bureau of Statistics (ICBS) report (Israel in Figures – Selected Data from The Statistical Abstract of Israel, 2019) [24], in 2018 a total of 88.9% of the Israeli population, lived either in cities (74.2%) or municipalities (14.7%) (page 30). The small population size vis-á-vis the concentration in densely populated areas enabled Israel agility and maneuverability in vaccine purchasing, as well as minimization of the transport and storage challenges associated with the Pfizer-BioNTech COVID-19 vaccine. Single state-of-the-art medical warehouse sufficed to store the nation’s entire Pfizer vaccine reserve in the requisite ultra-low-temperature freezers.

As our study indicates, in the absence of vaccination, the mortality growth rate is anticipated to rise with the city population. Yet, in the presence of vaccination, the mortality growth rate is anticipated to drop with the city population.

Based on Rosen et al. [11], other remarkable characteristics of Israel include:

1. Israel’s centralized national system of government (as opposed to a federal system of government). Public health-care issues are considered to be within the exclusive domain of the central government. Consequently, the national government had the primary responsibility for the vaccination campaign, and coordination efforts of a public health response across different levels of government were not required.
2. Israel’s experience in, and infrastructure for, planning and implementing prompt responses to large-scale national emergencies. As a result of its challenging geo-political position (the need to fight against external and internal threats) Israel developed an “all hazards” approach and invested substantially in preparing for large-scale emergencies, whether they be related to security, natural disasters, or health.
3. The organizational and logistic capacities of Israel’s community-based healthcare providers (the four health plans), which are all large and national in scope. To address inequalities in availability and access to health care, legislation providing for universal health-care insurance for all Israeli citizens was passed in 1995 [12]. This law provides a broad basket of high-quality preventive, curative, and rehabilitation health-care services, as well as universal national health insurance coverage, financed primarily through income-related tax revenues. All permanent residents are free to choose from among Israel’s four large, competing, nonprofit, health plans.
4. The availability of a cadre of well-trained, salaried, community-based nurses who are employed directly by the health plans. Many of the nurses, employed by the community-based healthcare providers, have experience administering vaccinations, making it relatively easy for the plans to shift some of them from their regular tasks to the COVID-19 vaccination effort. These are skilled and well-trained professionals who could start vaccinating immediately.

One of the limitations of this study is the fact that the error term is not normally distributed. This may be further corroborated based on qqplot graphs (using the qnorm and pnorm commands in Stata software package). The potential concern is that the standard errors of the parameters are biased and inconsistent. An alternative way to address this problem is the use of robust standard errors. This robustness test yields similar graphs to those reported in Figs. 2, 3, 4.

## Conclusions

The objective of the current study is to investigate the projected daily COVID19 mortality growth rate with increased median population age and size of cities under two scenarios: with and without the BNT162b2 Pfizer vaccination against the SAR-COV2 virus. This study employs a panel dataset. We follow the COVID19 mortality growth rate in each of the 173 Israeli municipalities starting from March 21, 2020 (10 days after the first documentation of COVID19 cases in Israel) until September 21, 2021, where the BNT162b2 Pfizer vaccinations were available, starting from December 20, 2020.

A unique feature of Israel is the early initiation of a nationwide vaccination campaign that resulted in the full vaccination (i.e., receipt of two vaccine doses) in more than half the population by the end of March 2021 (e.g., [10]. Consequently, Israel provides a natural experiment for the efficiency of the vaccination.

Referring to population size of cities, findings demonstrate that while under the scenario without vaccination, the daily mortality growth rate is anticipated to *rise*, under the scenario with vaccination, daily mortality growth rate is anticipated to *drop*. Given the elevated number of interactions in more crowded cities (e.g., [9, 17, 18], the vaccination against SARS-COV2 virus might prove to be particularly important in larger, denser cities.

Moreover, referring to the median age of residents in the municipalities, findings suggest that the BNT162b2 Pfizer vaccinations attenuate the pace of anticipated daily mortality growth rate *rise* for 30 years of median age (from 11 years, the minimum median age to 41 years, the maximum median age). The literature indeed demonstrates that age is a risk factor for COVID19 infection and mortality (e.g., [5, 15, 16].

The principal public policy repercussion of our study is the importance of increasing awareness by promoting vaccination campaigns in schools, in conferences and via on-line digital means, and allocating of public health experts to enhance communication channels with the public, particularly in crowded municipalities.^10^ Savulesco et al. [28] review different incentives to get vaccinated, including COVID19 and non-COVID19 examples. The disincentive part include: fines (in Indonesia following a refusal to get the COVID19 vaccination and Germany following a refusal of children to be vaccinated against measles), withholding state benefits (in Indonesia and Australia), avoiding accessibility to social goods (vaccination as a condition of entry to campus at some US colleges and enrollment in US public schools) and employment (service staff in England and Moscow can be prevented from entering or continuing in a professional role if they have not been vaccinated). The incentive component includes financial payment (in Serbia), cash lotteries (vaccinated people are entered into a lottery for a substantial cash prize in Ohio and Kentucky), investment opportunities in saving bonds for 16–35 years of age people if they get vaccinated in West Virginia and payments in kind, such as, free beer in Connecticut, or guns in West Virginia [28].

Referring to the Israeli context, according to [11], one of the remarkable features of Israel is the well-tailored outreach efforts to encourage the population to sign up for vaccinations (Rosen et al. [11]: page 4). Israel took a variaty of steps to promote vaccinations. One of which is a mass and a social media campaign following the rigorous review process and approval of the vaccination by the highly prestigious US FDA. Another is to create different channels through which people could schedule vaccinations, such as, phone calls, the website, applications of the four health care centers. A third step is the provision of daily updates on the number of vaccinated Israelis, accompanied by video clips and photos, thus creating a source of national pride and a reassurance to persons who might otherwise be hesitant. Finally, the Ministry of Health has invested substantial effort in recruiting the support of religious leaders of Ultra-Orthodox jews. Among the Haredi population, this bore fruit already in the first week of the vaccination campaign. (Rosen et al. [11]: page 10).

Other measures include: mobile vaccination units sent to Arabs residents of remote areas (periphery), Bedouins; and universities, beaches, and commercial streets populated by young adults with perception of limited personal benefits from vaccine; Appointment of coordinators to provide the need for child care during vaccination; Establishment of the Green Pass program which gives better accessibility to workplace and shopping centers; Development of Arabic-language materials and use of Arabic language media; Dissemination of information, including coverage of cases where pregnant women got severely ill and were not vaccinated; Partnering with community leaders [11].

Additional important public policy repercussions should include stratification of cities and neighborhoods by age and population. As demonstrated in Fig. 2, given the positive correlation between the median age and COVID-19 mortality growth rates, under equal conditions, the vaccination policy should prioritize cities with old population. Consequently, cities with the youngest population will be the last to receive the doses of vaccine, and cities with the oldest population will be the first to accept the doses of vaccine. Moreover, a city with a larger age variance should be prioritized with respect to cities with the same young median age and smaller age variance. Finally, as Fig. 3 demonstrates, given the negative (positive) mortality growth rate with population in the presence (absence) of vaccines, under equal conditions, the vaccination policy should prioritize more crowded cities.

Since age has a well-known prognostic effect both on infection and severity of COVID-19 morbidity, the analysis permits derivation of several principles for future potential pandemics.

In cities, where the median age is high, two perspectives of early and intensive public policy interventions are clearly required.

The first perspective is prevention via elevation of dedicated health services, establishment of testing and vaccination complexes; generating selective lockdowns; education for increasing awareness to social distancing, wearing masks and other preventive means.

The second perspective is extensive medical treatment, namely, extension of availability of medical physical and online services; dispensing designated medications; expansion of hospitalization facilities and information services particularly to susceptible populations. All measures will be taken with attention to age accessibility of these means. These measures may warrant a separate public policy study in preparation for dealing with future pandemics.

## Data Availability

The datasets used and/or analyzed during the current study are available from the corresponding author upon reasonable request.

## Appendix: Per-capita GDP in OECD countries

Source: OECD Data: Gross Domestic Product, available at: https://data.oecd.org/gdp/gross-domestic-product-gdp.htm (Last accessed on December 2, 2021) [26].

## Abbreviations

SARS-CoV-2: Severe acute respiratory syndrome coronavirus 2
COVID19: Coronavirus disease 2019
US: United States
GDP: Gross domestic product
OECD: The organization for economic co-operation and development
WHO: World Health Organization

## Footnotes

1 BNT162b2 vaccine is based on mRNA technology, which was familiar to medical researchers for more than a decade prior to the outbreak of the pandemic. This technology was previously developed to address pathogens that resemble the SARS-CoV2 virus, such as, SARS and MARS. Earlier work had suggested that the spike protein of the coronavirus responsible for the 2002 SARS outbreak was a suitable target for protective immunity [1] The development of this technology was accelerated following the COVID19 pandemic, with the prior familiarity with this technology enabling an abbreviated development period.

2 The 16 million deaths is the evaluation of the Economist for the number of dead persons until the end of the pandemic. Note, that due to the concern that the official counting missed the true extent of mortality from SARS-CoV2, an alternative way to measure the extent of COVID19 mortality is excess mortality, namely, a comparison between the number of deaths from all reasons before and after the outburst of the COVID19 pandemic.

3 For a comparative per-capita GDP ranking among OECD countries, see the graph in the “Appendix”.

4 These precentages are calculated as one minus the ratio between the number of new cases among the vaccinated / unvaccinated group. Referring to documeneted infections, for example, Fig. 2 in Dagan et al. [14] demonstrates that among the vaccinated group, the documented number of new cases 14–21 days after the first dose is 591 (= 4124 − 3533). The equivalent figure among the unvaccinated group is 1,133 (= 5104 − 3971). Substitution in the formula yields: $\left(1-\frac{591}{\mathrm{1,133}}\right)=47.83\%\approx 46\%$.

5 The constant growth rate model is given by${Y}_{t}={Y}_{0}{(1+g)}^{t}$. Taking the natural logarithm yields: $ln{Y}_{t}=\alpha +\beta t$ where $\alpha =ln{Y}_{0}$ and $\beta =\mathrm{ln}\left(1+g\right).$ If the model is given by $ln{Y}_{t}=\alpha +\beta t$, then$\Delta ln{Y}_{t}=\beta =\mathrm{ln}(1+g)\approx g$, namely, an approximation of the discrete growth rate: “Thus, taking first differences of logs gives the continuous growth rate, which in turn is an approximation to the discrete growth rate. This approximation is only reasonably accurate for small values of *g*.” [19], 43 at the bottom). The precise discrete growth rate (*g*) is given by: exp(*b*) − 1, where *b* is the estimated *β*.

6 The product of the average population size (51,215) and the number of municipalities ($i=173$) yields 8,860,195 persons. According to Israeli Central Bureu of Statistics media release from December 31, 2021, the population in Israel is 9,291 million persons. Consequently, the sample covers $\frac{\mathrm{8,860,195}}{\mathrm{9,291,000}}=95.36\%$ of the Israeli population.

7 The transformation (*MedianAge*_*i*_ − 11) is based on Ramanathan [20]: 147–148 (the constant term as a conditional forecast). The reason for using the transformation (*MedianAge*_*i*_ − 11) is that the minimal median age in the sample is 11 years. If we put *MedianAge*_*i*_ instead of (*MedianAge*_*i*_ − 11), the constant term (the baseline mortality growth rate) will forecast an out of sample city with a median age of babies, all of whom were recently born. Given that the Population size and population density are close to zero, this problem does not exist for these explanatory variables.

8 See, for example: Israel Central Bureau of Statistics (ICBD): Media Release 155/2021: Selected Data on the Occasion of Jerusalem Day. Available at: https://www.cbs.gov.il/en/mediarelease/Pages/2021/Selected-Data-on-Jerusalem-(2018-2020).aspx (Last accessed on December 2, 2021) [21, 22].

9 According to Kmenta [23]: “Another possible explanation of a low value of *R*2 is that while *X* is the relevant explanatory variable, its influence on Y is weak compared to the influence of the random disturbance. This indeed seems to be the case for relationships describing household behavior that have been estimated from cross-section data. For example, a typical *R*2 for various household behavior function from the University of Michigan’s Survey Research Center data is close to 0.20.” (page 242).

10 Smallpox is a unique example of a lethal pandemic that was eradicated by global vaccinations program. Of the seven attempts so far made at global eradication of human infectious diseases, only one the eradication of small pox has succeeded [29]. According to Fenner et al. [30] vaccination against smallpox had been practiced in virtually every country of the world, and in many on a large scale, when the Intensified Smallpox Eradication Program was launched in 1967. (Page 540) Smallpox was declared eradicated 40 years ago, in 1980, after unparalleled devastation of human populations for many centuries. Until the nineteenth century, smallpox is thought to have accounted for more deaths than any other single infectious disease, even plague and cholera. In the city of London, England alone, more than 320,000 people are recorded to have died from smallpox since 1664 [25]
